# Risk factors for acquisition of carbapenem-resistance during treatment with carbapenem in the intensive care unit: a prospective study

**DOI:** 10.1007/s10096-019-03644-6

**Published:** 2019-09-03

**Authors:** François Labaste, Julia Grossac, Fanny Vardon Bounes, Jean-Marie Conil, Stéphanie Ruiz, Thierry Seguin, Marion Grare, Olivier Fourcade, Vincent Minville, Bernard Georges

**Affiliations:** 1 Service de Réanimation Polyvalente, CHU Rangueil, 1 Avenue Jean Poulhès, Pôle d’Anesthésie et Réanimation, TSA 50032, 31059 Toulouse Cedex 9, France; 2 Laboratoire de Bactériologie et Hygiène, Institut Fédératif de Biologie, 330 Avenue de Grande Bretagne, TSA 40031, 31059 Toulouse Cedex 9, France; 3 Department of Anaesthesiology and Intensive Care Units, University Hospital of Toulouse, 31059 Toulouse Cedex 9, France

**Keywords:** Carbapenem resistance, *Pseudomonas aeruginosa*, Carbapenemase

## Abstract

The emergence of carbapenemases in gram-negative aerobes is worrying. The aim of this prospective study was to estimate the incidence of acquisition of carbapenem-resistance during treatment in ICU and to identify the risk factors. This was a prospective, observational, cohort study. This study was conducted at intensive care unit, academic medical center, Toulouse Rangueil University Hospital. Patients were included if they received antibiotic treatment with carbapenem for more than 48 h. Biological samples were taken in accordance with current practice in the unit. The main endpoint was the occurrence of bacterial resistance to carbapenems occurring between the onset of treatment and the patient’s exit from the ICU. Uni- and multi-variate analyses were carried out. Of the 364 patients admitted to the unit between May and November 2014, 78 were included in our study and 16 (20.51%) developed resistance. The two main risk factors were a length of stay in ICU of more than 29 days (HR = 3.61, *p* = 0.01) and the presence of *Pseudomonas aeruginosa* in the samples taken before the start of treatment (HR = 5.31, *p* = 0.002). No resistance due to carbapenemase production was observed in this study. The prescription of carbapenems in the ICU setting must adhere to the expert guidelines. In light of our results, special attention must be paid to patients whose stay in intensive care is prolonged, and those in whom *Pseudomonas aeruginosa* is isolated from bacteriological samples taken before the beginning of antibiotic therapy.

## Introduction

The widespread use of beta lactams, particularly in the intensive care unit (ICU) setting, has led to the occurrence of bacterial resistance. Indeed, the emergence and spread of new beta-lactamases, the principal mechanism involved in bacterial resistance of gram-negative bacteria, closely paralleled the sequential introduction of different beta lactams [1]. Over the last decade, the prevalence of gram-negative bacterial pathogens resistant to multiple antibiotics has dramatically increased [2] and extended-spectrum beta lactamases (ESBLs) are now common in intensive care units.

Gram-negative bacterial (GNB) infections are serious and life-threatening. Any delay in management, or inadequate antibiotic therapy, can have serious consequences in terms of prognosis. To save the patient, an effective antibiotic therapy must be promptly initiated [3].

Carbapenems have a broad spectrum of activity, and have high stability with respect to almost all the beta-lactamases. Carbapenems exert their bactericidal activity by binding to penicillin-binding proteins, PLPs, in particular PLP1a, 1b, and 2, resulting in lower endotoxin release during lysis of gram-negative bacilli. Thus, they are often the antibiotics of choice in the ICU for nosocomial infections [4–8].

Nevertheless, carbapenem resistance among gram-negative pathogens has been reported worldwide [1]. In the case of Enterobacteriaceae and *Pseudomonas aeruginosa*, resistance to carbapenems is mainly due to a combination of mechanisms, often involving the production of beta-lactamases as well as defective or damaged porins [1].

The emergence of carbapenemases in gram-negative aerobes also is worrying [1, 9, 10]. In recent years, strains of carbapenemases-producing enterobacteria have been observed [1, 10]. These carbapenemases may be encoded by chromosomal genes or plasmid metallo-enzyme genes. In the strains of carbapenemase-producing enterobacteria which have appeared in several countries, including Greece, Israel, and France, the genetic origin is plasmid-based, and therefore transmissible.

Expert recommendations on the use of carbapenems attempt to limit their prescription in order to maintain their effectiveness [11] and prevent the emergence of widespread resistance which would result in deaths due to lack of effective antibiotics [12]. These recommendations have been formalized by experts to guide the adequate use of carbapenems [13]. To limit carbapenem use, practitioners should reserve this antibiotic for patients at high risk of ESBL-producing GNB. The main risk factor for carbapenem-resistance is prior use of carbapenems. Many other factors have been described, including previous piperacillin–tazobactam treatment and its duration, ventilator-acquired pneumonia, and presence of intravascular devices, but the relative importance of each remains unclear. There have been few prospective studies on this topic and risk factors are still poorly understood.

The aim of this prospective study was to estimate the incidence of acquisition of carbapenem-resistance during treatment in ICU and to identify the risk factors involved.

## Methods

### Setting

This prospective, observational, cohort study was conducted from November 2014 to May 2015 in the ICU of Rangueil Hospital in Toulouse, France, in a 24-bed ICU which admits surgical and medical patients. Surgical patients included those undergoing vascular, urological, or digestive surgery if ICU care was needed 1 day after the operation. Patients with cardiac surgery were managed in a specialist unit and secondarily transferred to our service in case of complications.

The protocol was approved by the local Research Ethics Committee (no. 64-1212). This IRB waived the need for consent.

### Study design and data collection

#### Inclusion and exclusion criteria

Consecutive patients aged over 18 years, having received at least 48 h of carbapenem therapy were included. Treatment could be imipenem, doripenem, or meropenem.

The following were excluded: patients with a stay on ICU of less than 48 h, those who had received carbapenem therapy for less than 48 h, or were on ertapenem treatment, minors or persons under legal protection, and patients for whom a decision had already been taken to limit or withdraw active treatment upon inclusion, and who did not take bacteriological samples.

An infection was defined by a positive culture, quantitatively significant, and local or general signs of infection meeting the criteria in effect [14].

### Data collection

After inclusion, the following demographic data were collected: age, reason for admission, past medical history, mechanical ventilation duration, ICU stay, and ICU mortality. The illness severity was evaluated by the SAPS-2 scoring system.

All antibiotic treatments received up to 1 month prior to admission in our unit were identified. Antibiotics given after inclusion, including carbapenems, were also identified. For each of these antibiotics, the variables collected were: indication, duration and delay between admission, and initiation of the treatment.

For the duration of antibiotic treatment with carbapenem, we recorded the total duration of antibiotic therapy, as well as the duration of antibiotic therapy until the onset of resistance. The latter was defined by the duration between the start of treatment and the end of treatment if no resistance occurred during the treatment period, or by the duration between the start of treatment and the date of occurrence of carbapenem resistance during the treatment period.

Prescriptions of antibiotics are carried out by seniors and on the basis of written protocols. Within the team, there is a referent in antibiotic therapy present every morning during transmissions. Once a week, an infectious disease meeting brings together the anesthesiologist, an infectious disease consultant and a clinician pharmacist.

### Bacteriological testing

Bacteriological samples, as tracheal aspiration or bronchoalveolar lavage, were obtained in accordance with standard practice in the unit. Nasal and anal margin and respiratory sampling was performed on admission and repeated once per week.

In cases of sepsis or fever, we routinely took aerobic and anaerobic blood cultures and bronchial samples. If needed, samples were also taken from operative sites, drains, and central catheters.

### Clinical assessment and outcome measurement

The primary endpoint was the occurrence of carbapenem resistance, diagnosed between the beginning of carbapenem therapy and the patient’s discharge from the ICU. In cases where several bacterial strains became resistant, we only considered the first.

We also checked the appropriateness of the antibiotic prescription in relation to expert recommendations. This criterion was checked specifically at day 3 of treatment, the time at which a de-escalation should be considered. If the prescription conformed to expert guidelines, it was validated as a relevant requirement. The mortality considered was that which occurred only during the hospitalization in ICU.

### Statistical analysis

For descriptive statistics results were expressed as median and 95% CI. The study population was then divided into 2 groups based on the occurrence of carbapenem resistance after a period of up to 90 days.

Patient characteristics were compared using non-parametric tests (Mann-Whitney *U* test for continuous variables and Fisher’s exact test for categorical variables).

We evaluated the performance of each covariate as a predictor of resistance development by studying the receiver operating characteristic (ROC) curves and subsequently comparing the associated area under the curve (AUC). The most discriminating thresholds were chosen according to the optimum Youden index.

The association between the different covariates and the dependent variable (resistance to carbapenems) was calculated by multivariate analysis, measuring the hazard ratio (setting an initial threshold at *p* < 0.2). Statistically, this information was censored since it was not known on what date a subject could have acquired carbapenem resistance had he/she been followed up for a longer time. We therefore used a Cox survival model, employing a methodology described in a previous study [15]. After exclusion of collinear covariates, stepwise regression (backward elimination) was applied, starting with all the variables initially chosen and then progressively removing non-significant ones. Several models were tested, taking into account the limited number of events that limited the number of variables to be included in each model.

We used the MedCalc® Version 15 statistical software (Mariakerke, Belgium). A *p* value of < 0.05 was considered statistically significant.

## Results

### Patients and clinical characteristics

**Fig. 1:**
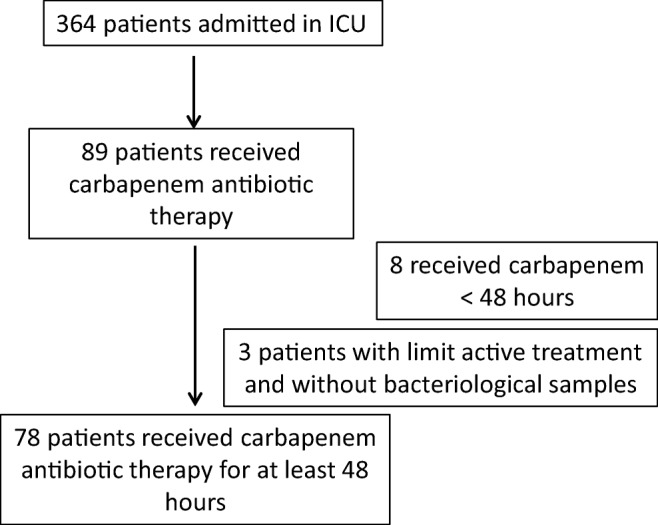
Flowchart of inclusions

From May to November 2014, 364 patients were admitted to our unit. Among them, 78 received carbapenem antibiotic therapy for at least 48 h and were therefore included in the study (Fig. 1). Meropenem was the most widely prescribed antibiotic, with 76 patients (97.5%) benefiting from it. Imipenem was only used in 2 patients (2.5%). The most common reason for admission was polytraumatism or vascular, urological, or digestive post-surgery (57.7% of cases).

Patient characteristics are displayed in Table 1. 74.4% were men, the median age was 62.5 years, and the mortality rate was 32.1%. Carbapenem treatment was prescribed due to ventilator-associated pneumonia (34.6%), community-acquired pneumonia, peritonitis, and bacteraemia (Table 2). In 78.2% of cases, it was empirical therapy. There was no difference among patients who died of septic shock between those in whom resistance appears compared to the control group.

**Table 1:** General characteristics of the population (*n* = 78)

	Median	95% CI
Age (years)	62.5	58–66
Weight (Kg)	77	73–82.1
Height (cm)	171.5	167–175
SAPS2 score	52.5	45–58
Duration of hospitalization on day of inclusion (days)	10.5	9–14
Duration of prior antibiotic therapy (days)	8	6–10
Duration of carbapenem antibiotic therapy (days)	8	6–9
Duration of antibiotic therapy up to the onset of resistance (days)	7.5	6–9
Duration of hospitalization in intensive care unit (days)	16	10.9–20.1
Total mechanical ventilation time (days)	11	6–16.1
Duration of mechanical ventilation before inclusion (days)	1.5	0–4
Total duration of catecholamine treatment (days)	4	3–8

Data are expressed as median (confidence interval 95%)

**Table 2:** Acquired carbapenem resistance: univariate analysis, categorical variables

	Total	No resistance (*n* = 62)	Resistances (*n* = 16)	*p*
Males, *n* (%)	58 (74.4%)	45(72.6%)	13 (81.2%)	0.75
Medical history, *n* (%)				
Chronic obstructive pulmonary disease	16 (20.5%)	13 (21.0%)	3 (18.8%)	1
Respiratory failure	13 (16.7%)	11 (17.7%)	2 (12.5%)	1
Chronic heart failure	18 (23.1%)	16 (25.8%)	2 (12.5%)	0.34
Myocardial infarction	21 (26.9%)	19 (30.6%)	2 (12.5%)	0.21
Atrial fibrillation	11 (14.1%)	8 (12.9%)	3 (18.8%)	0.69
Arterial hypertension	37 (47.4%)	30 (48.4%)	7 (43.7%)	0.79
Neoplasia	20 (25.6%)	16 (25.8%)	4 (25%)	1
Alcohol	16 (20.5%)	14 (22.6%)	2 (12.5%)	0.50
Admission pattern, *n* (%)				
Medical	31 (39.7%)	25 (40.3%)	6 (37.5%)	1
Polytrauma and postoperative patients	45 (57.7%)	35 (56.5%)	10 (62.5%)	0.78
Type of infection, *n* (%)				
Ventilator-associated pneumonia	27 (34.6%)	21 (33.9%)	6 (37.5%)	0.78
Community acquired pneumonia	12 (15.4%)	10 (16.1%)	2 (12.5%)	1
Peritonitis	9 (11.5%)	5 (8.1%)	4 (25.0%)	0.08
Bacteraemia	14 (17.9%)	12 (19.4%)	2 (12.5%)	0.8
Data relating to stay in intensive care unit, n (%)				
Tracheotomy	14 (17.9%)	9 (14.5%)	5 (31.2%)	0.15
ARDS	12 (15.4%)	8 (12.9%)	4 (25.0%)	0.25
Non-invasive ventilation	23 (29.5%)	16 (25.8%)	7 (43.7%)	0.22
Septic shock	43 (55.1%)	35 (56.5%)	8 (50.0%)	0.78
Prior antibiotic therapy	70 (89.7%)	54 (87.1%)	16 (100%)	0.20
*Piperacillin-tazobactam*	49 (62.8%)	37 (59.7%)	12 (75.0%)	0.39
*Aminoglycoside*	47 (60.3%)	36 (58.1%)	11 (68.7%)	0.57
*Fluoroquinolone*	13 (16.7%)	10 (16.1%)	3 (18.8%)	0.72
Deaths in intensive care unit, *n* (%)	25 (32.1%)	21 (33.9%)	4 (25%)	0.56
Death by septic shock, *n* (%)	43 (55.1%)	9 (25.7%)	16 (37.2%)	0.33
Adequacy criteria, *n* (%)				
Empirical medical prescription	62 (79.5%)	51 (82.3%)	11 (68.7%)	0.30
Therapeutic de-escalation	20 (25.6%)	16 (25.8%)	4 (25%)	1
Re-evaluation during treatment	57 (73.1%)	43 (69.4%)	14 (87.5%)	0.21
Appropriateness	55 (70.5%)	42 (67.7%)	13 (81.2%)	0.37
Bacteriological data, *n* (%)				
Presence of *P. aeruginosa* before inclusion	17 (21.8%)	9 (14.5%)	8 (50.0%)	0.004

Data are expressed as median (confidence interval 95%) or counts (percentage). It is significant with *p* lower than 0.05

We noticed the duration of hospitalization on day of inclusion (10.5 days as median), the duration of prior antibiotic therapy (8 days as median), the duration of carbapenem antibiotic therapy (8 days as median), the duration of antibiotic therapy up to the onset of resistance (7.5 days as median), the duration of hospitalization in intensive care unit (16 days as median), and the total mechanical ventilation time (7.5 days as median).

### Carbapenem resistance

Sixteen patients (20.5%) developed carbapenem resistance while on treatment with this antibiotic. The 2 patients treated with imipenem acquired a resistance by modification of the outer membrane protein OprD. Patients treated with meropenem acquired a resistance by impermeability with overproduction of efflux system alone or associating the outer membrane protein OprD. None resistance due to carbapenemase production was observed.

The bacterial strains were: 14 *Pseudomonas aeruginosa*, 1 Pseudomonas putida, and 1 ESBL *Citrobacter sedlakii*. No carbapenemase was found. During this study, one patient was admitted with infection due to ESBL Klebsiella pneumonia, carrying a carbapenemase upon entry into the service. This patient was not included in the study.

### Risk factors for acquisition of carbapenem resistance

The univariate analysis is shown in Table 3 and Table 4. Risk factors were duration of mechanical ventilation (*p* = 0.04), duration of stay in ICU (*p* = 0.001), and duration of carbapenem treatment (*p* = 0.04).

**Table 3:** Acquired carbapenem resistance: univariate analysis, continuous variables

	No resistance (*n* = 62)		Resistance (*n* = 16)		
	Median	95% CI	Median	95% CI	*p*
Age (year)	63.0	58.8–66	60	51.6–77.4	0.91
Weight (Kg)	79.5	74.8–86	71.5	68.2–82.4	0.11
Height (cm)	172	167–175	170	161.8–175.5	0 .53
SAPS2 score	54.5	49.4–62	43	30.6–57.4	0 .09
Duration of hospitalization before the inclusion (days)	1	0–3.2	2.5	1–10.4	0 .08
Duration of prior antibiotic therapy (days)	7.5	5.8–10	8.5	2.64–21	0.40
Duration of carbapenem antibiotic therapy (days)	7	5–9	9.5	7.3–11.4	0.04
Duration of antibiotic therapy up to the onset of resistance (days)	7.0	5.0–9.0	8.5	6–10.4	0.20
Duration of hospitalization in intensive care unit (days)	12	8.8–17	33	20.5–42.7	0.001
Total mechanical ventilation time (days)	8.5	6–15	25	5.3–35.6	0.04
Duration of mechanical ventilation before inclusion (days)	1	0–4.2	2.5	0.64–8.72	0.35
Duration of catecholamine treatment (days)	4	3–8	10.5	2. - 22.4	0.24

Data are expressed as median (confidence interval 95%) or counts (percentage). It is significant with *p* lower than 0.05

**Table 4:** Comparison of areas under the curve for continuous variables predicting the acquisition of carbapenem resistance

	AUC	95% CI	threshold	Se%	CI 95%	Sp. %	95% CI	PPV	NPV
Duration of treatment with carbapenem	0.664	0.55–0.77	> 7	75	47–92.7	54.8	41.7–67.5	30	89.5
Duration of hospitalization in intensive care unit	0.761	0.65–0.85	> 29	68.7	41–89	80.6	69–90	47.8	90.9
Duration of mechanical ventilation	0.669	0.55–0.77	> 20	62.5	35–85	75.8	63–86	40	88.7

*AUC* area under the curve, *95% CI* 95% confidence interval, *Se* sensitivity, *Sp* specificity

*PPV* Positive predictive value (probability of resistance at 3 months when the test is positive)

*NPV* Negative predictive value (probability of sensitivity at 3 months when the test is negative)

The presence of a *Pseudomonas aeruginosa* strain in bacteriological samples was associated with risk of carbapenem resistance (*p* = 0.004).

Time between start of carbapenem treatment and the occurrence of carbapenem resistance did not affect the risk (*p* = 0.20).

**Fig. 2:**
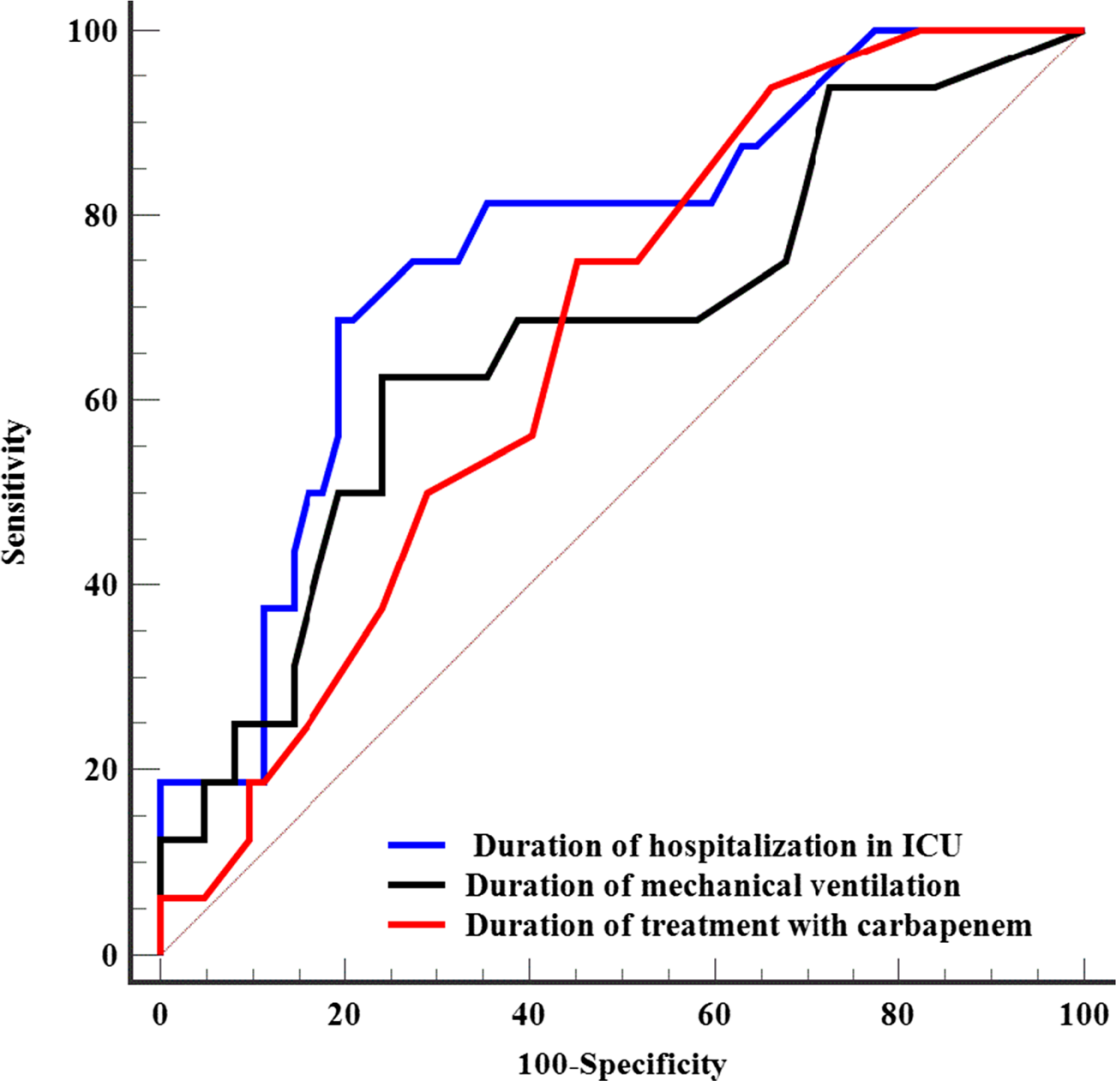
Comparison of ROC curves. The ROC curves showed that the AUC for ICU length of stay was 0.76, with an optimal threshold of > 29 days

On multivariate analysis, a total ICU stay duration of greater than 29 days was associated with acquisition of carbapenem resistance (HR = 5.31; *p* = 0.002). The ROC curves showed that the AUC for ICU length of stay was 0.76, with an optimal threshold of > 29 days (Fig. 2). Presence of *Pseudomonas aeruginosa* in bacteriological samples before treatment was also an independent risk factor (HR = 3.61; *p* = 0.01). Results are shown in Table 5.

**Table 5:** Multivariate Cox survival analysis identifying variables independently associated with emergence of carbapenem resistance

	HR (95% CI)	*p*
*Pseudomonas aeruginosa* in bacteriological samples before treatment	3.61(1.35–9.67)	0.01
ICU duration of stay greater than 29 days	5.31(1.8–15.37)	0.002

*HR* hazard ratio, *CI* confidence interval

### Adequacy of prescription

Table 2 shows the criteria of adequacy of antibiotic prescription. Adequacy refers to the recommendations of experts in terms of appropriateness, good concentration, and good route of administration [13]. Carbapenem treatment was appropriate in 70.5%. Inappropriate prescription was not associated with acquisition of carbapenem resistance.

## Discussion

In this study, we found two independent risk factors for acquisition of carbapenem resistance: a length of stay in ICU of greater than 29 days, and the presence of *Pseudomonas aeruginosa* in bacteriological samples taken before treatment.

Ours is one of the few prospective studies focusing on risk factors for development of bacterial resistance to carbapenem. Epidemiological data on the acquisition of carbapenem resistance have mainly been based on retrospective case-control studies [16–18] and previous prospective studies [19, 20] have used different designs. Inclusion criteria were only admission to an ICU, whereas we choose initiation of carbapenem therapy. Exposure to antibiotics, including carbapenem, was the main risk factor reported [21, 22]; hence, we focused specifically on patients exposed to these antibiotics.

Some authors have considered the *Stenotrophomonas maltophilia* colonization/infection as a factor contributing to acquisition of bacterial resistance [20, 21]. Given the natural resistance of *S. maltophilia* to carbapenems, we considered positive samples to be the result of a simple selection process and, hence, did not take them into account in our study.

We did not include the patients with carbapenem for less than 2 days, because in our department, we regularly monitor the risk of developing resistance to several antibiotics, including carbapenems. This risk is rare in our experience and did not affect the 8 excluded patients.

In our population, 20.5% of patients acquired bacterial resistance after initiation of carbapenem treatment. The incidence of carbapenem resistance is on the increase. For example, one Greek study in 2006 found an incidence of 13% [20], and in 2011, a French study reported acquisition of carbapenem resistance in 6.6% of patients admitted to the ICU [21]. This increase is mainly explained by a rise in the number of cases with carbapenem resistant bacteria [1], particularly *Pseudomonas aeruginosa* strains [22]. According to the EARSS network in France, carbapenem resistance of *Pseudomonas aeruginosa* isolated from blood cultures rose from 12.2 to 17.4% between 2006 and 2009 [1]. In 2014 (the period covered by our study), the risk of developing resistant bacterial infection was 20.5%. However, our population was different. Only patients treated with carbapenems were included, and carbapenem exposure is a risk factor in itself [23]. Our population therefore had a higher risk than the overall ICU population, and the true incidence might have been slightly lower. Also, our institution only uses meropenem, so, even though, according to literature, meropenem is more effective than imipenem [24], comparison with the EARSS report [1] is difficult. Of note, meropenem could intrinsically be associated with a larger increase in resistance than imipenem, due to its effect on microbiota [25]. Furthermore, the mechanism for carbapenem resistance in our study was never involved carbapenemase production. The main mechanism of resistance to imipenem in *P. aeruginosa* is well known and remains due to a modification of the outer membrane protein OprD [4, 15]. Resistance to carbapenems may result from combined mechanisms of resistance associating β-lactamases and decreased outer membrane permeability, or from carbapenemases as KPC-type enzymes, metallo-β-lactamases, or oxacillinase as OXA-48 [1]. Resistance to meropenem can potentially be due to overproduction of the MexAB-OprM efflux system [26].

The length of stay in ICU emerged as a predominant risk factor. This factor has also been found in other studies, highlighting the need for strict control of this class of antibiotics when used in patients who have a prolonged stay in ICU [27, 28].

Similarly, use of a carbapenem must be carefully controlled in patients who are colonized or infected with *Pseudomonas aeruginosa* (adjusted HR = 3.61, 95% CI 1.35 to 9.67 2.38, 95% CI 1.53 to 3.69) [12]. Indeed, we showed that presence of *Pseudomonas aeruginosa* is a major risk factor for developing carbapenem resistance.

In our study, we did not find any impact on mortality. No deaths of patients included in our study are related to the impossibility of antibiotic treatment by multidrug resistance. Also, in contrast to other studies, prior exposure to other antibiotics, including tazocillin or aminoglycoside, was not correlated with risk of carbapenem resistance [19–21]. As a result of the current guidelines, carbapenems are rarely used as the first-line antibiotic; hence a large number of patients will have received therapy with other antibiotics [11, 13]. In our study, for example, 89.7% of patients had received such prior antibiotic therapy.

In the univariate analysis, the duration of carbapenem therapy also appeared as a risk factor for development of resistance. The relevance of this data however remains questionable. In fact, some patients acquire resistance while the antibiotic is ongoing. Paradoxically, when we considered the duration of carbapenem therapy up to the time resistance was diagnosed, this did not appear to be significant. The number of patients was probably too small to demonstrate this effect.

Compliance of antibiotic prescription with the recommendations did not appear to be a risk factor for acquisition of resistance. 70.5% of prescriptions were considered appropriate, which is comparable with data from other groups [29].

This study has several limitations. Firstly, it is a single-center study. Bacterial ecology is ICU dependent, so results should be interpreted with caution. Secondly, the number of patients remains relatively small. Finally, some patients might have developed bacterial resistance to carbapenems which we failed to diagnose.

## Conclusion

A length of stay in ICU of greater than 29 days, and the presence of *Pseudomonas aeruginosa* in bacteriological samples before treatment were the two independent risk factors for acquisition of carbapenem resistance in this study. Resistance due to carbapenemase production was not observed.

Patients are at risk of developing bacterial resistance to carbapenems, and prescription of these antibiotics in the ICU must adhere to the expert guidelines. Particular attention should be paid to patients with an extended stay in ICU and to patients with samples positive for *Pseudomonas aeruginosa*.

### Contributions of the authors

Conception and design: BG, FL, JMC, VM, MG; analysis and interpretation: BG, FB, SR, TS; editing the manuscript for important intellectual content: BG, OF, JG.

### Availability of data and material

All the samples were sent to the Laboratory of Bacteriology and Hygiene, Federative Institute of Biology of our institution. The MICs of the antibiotics were examined in this unit. Data can be found in our intensive care unit or in the Laboratory of Bacteriology and Hygiene.
